# Coarse-Grained Conformational Sampling of Protein Structure Improves the Fit to Experimental Hydrogen-Exchange Data

**DOI:** 10.3389/fmolb.2017.00013

**Published:** 2017-03-10

**Authors:** Didier Devaurs, Dinler A. Antunes, Malvina Papanastasiou, Mark Moll, Daniel Ricklin, John D. Lambris, Lydia E. Kavraki

**Affiliations:** ^1^Department of Computer Science, Rice UniversityHouston, TX, USA; ^2^Department of Pathology and Laboratory Medicine, University of PennsylvaniaPhiladelphia, PA, USA; ^3^Broad Institute of MIT & HarvardCambridge, MA, USA; ^4^Department of Pharmaceutical Sciences, University of BaselBasel, Switzerland

**Keywords:** protein conformational sampling, coarse-grained conformational sampling, molecular dynamics, experimental data fitting, hydrogen/deuterium exchange, mass spectrometry, nuclear magnetic resonance spectroscopy, X-ray crystallography

## Abstract

Monitoring hydrogen/deuterium exchange (HDX) undergone by a protein in solution produces experimental data that translates into valuable information about the protein's structure. Data produced by HDX experiments is often interpreted using a crystal structure of the protein, when available. However, it has been shown that the correspondence between experimental HDX data and crystal structures is often not satisfactory. This creates difficulties when trying to perform a structural analysis of the HDX data. In this paper, we evaluate several strategies to obtain a conformation providing a good fit to the experimental HDX data, which is a premise of an accurate structural analysis. We show that performing molecular dynamics simulations can be inadequate to obtain such conformations, and we propose a novel methodology involving a coarse-grained conformational sampling approach instead. By extensively exploring the intrinsic flexibility of a protein with this approach, we produce a conformational ensemble from which we extract a *single* conformation providing a good fit to the experimental HDX data. We successfully demonstrate the applicability of our method to four small and medium-sized proteins.

## 1. Introduction

Hydrogen/deuterium exchange (HDX) is a chemical phenomenon in which hydrogen atoms of molecules are exchanged with deuterium atoms of the solvent (Engen et al., 2011). Contrary to other structural biology techniques, such as nuclear magnetic resonance (NMR) spectroscopy or X-ray crystallography, HDX experiments cannot reveal the three-dimensional structure of a molecule, but they can provide valuable structural information (Huang and Chen, 2014). This has led to numerous applications for the analysis of protein structure and conformational changes, as well as protein folding and interactions (Pirrone et al., 2015). As they monitor HDX over time (see Section 2.1), HDX detected by mass spectrometry (HDX-MS) experiments also allow studying protein dynamics (Wei et al., 2013). HDX-MS has benefited from the development of various computational tools (Claesen and Burzykowski, 2016), and has proven useful in the study of challenging systems, such as molecular complexes or membrane proteins (Harrison and Engen, 2016). Additionally, HDX-MS is having a deep impact in drug discovery and drug development (Deng et al., 2016), where it has helped characterize various biopharmaceuticals (Pirrone et al., 2015) and innate immunity proteins (Schuster et al., 2007; Sfyroera et al., 2015; Papanastasiou et al., 2017), among others.

Despite the clear benefits of monitoring HDX for structural analysis, it is sometimes difficult to interpret experimental HDX data. This data may be reported as protection factors (Jaswal, 2013), often visualized on a protein *heat map* (Huang and Chen, 2014) built using a structural model reported in the Protein Data Bank (PDB, RRID:SCR_012820), if available. However, it has been suggested that the correspondence between these structural models and experimental HDX data can be inadequate, especially for models produced by X-ray crystallography (Radou et al., 2014). This is due to the difference in nature between HDX data and crystallographic data: only HDX data can reflect the inherent variability of a specific protein state. As a result, it has been argued that experimental HDX data should rather be interpreted using a conformational ensemble produced by a molecular dynamics (MD) simulation (Best and Vendruscolo, 2006; Radou et al., 2014). However, this method can also fail at expressing the variability of a protein state in the same way as experimental HDX data does. In a previous study, we have observed that a single conformation extracted from a conformational ensemble produced by an MD simulation could provide a better fit to experimental HDX data than the whole ensemble (Devaurs et al., 2016). Therefore, it is reasonable to try and fit experimental HDX data using a single protein conformation; this can also be computationally advantageous.

In this paper, we propose a novel methodology to obtain a single conformation providing a good fit to the experimental HDX data collected for a protein, after confirming that crystal structures and conformations produced by MD simulations might not be good choices. Our methodology involves a coarse-grained conformational sampling tool that allows exploring the flexibility of a protein by generating a conformational ensemble, starting from the crystal structure of this protein (see Section 3.3). We evaluate our methodology on four small and medium-sized proteins that correspond to two scenarios: for three proteins, both the HDX data and the crystal structure are known to describe their native state; for one protein, the HDX data and crystal structure are known to describe two different states (see Section 3.4). The evaluation results show that our methodology can successfully produce conformations that provide a good fit to the experimental HDX data, for these four proteins (see Section 4).

A critical element of any method aiming to analyze the correspondence between a protein's structure and its HDX data is the definition of an *HDX prediction model*. Indeed, in such a method, some HDX data has to be derived from the protein's structure; then, one can assess the goodness-of-fit between this structurally-derived HDX data and the experimentally-observed HDX data. By comparing different protein conformations, it is then possible to determine which conformation provides the best estimates for the experimental HDX data (see Section 3.3). The challenge here is that, although numerous HDX prediction models have been proposed, none of them has yet been widely recognized and adopted by researchers in this field (see Section 2.2). Furthermore, a recent evaluation study has shown the limitations of several existing models (Skinner et al., 2012b). To mitigate this issue, we have integrated in our methodology the model that performed best in that evaluation study (see Section 3.1). Our approach compensates for the current limitations and achieves a successful application of this HDX prediction model (see Section 5). This is accomplished by using coarse-grained conformational sampling as a way to extensively explore the intrinsic flexibility of a given protein.

## 2. Background

### 2.1. Hydrogen/deuterium exchange (HDX) in proteins

Hydrogen exchange is a chemical phenomenon in which hydrogen atoms of proteins are exchanged with hydrogens in the surrounding solvent (Engen et al., 2011). Intuitively, the extent to which different parts of a protein are subjected to this exchange is influenced by their solvent accessibility and by the protein's structure (Wei et al., 2013). Therefore, researchers have worked on quantifying hydrogen exchange, as a way to gain information on a protein's structure. This is made possible by the fact that this exchange takes place with any isotope of hydrogen, such as deuterium. If a protein, initially kept in a regular water solution (H_2_O), is placed in a “heavy water” solution (D_2_O), the hydrogen in the protein will exchange with the deuterium in the solvent. This phenomenon is referred to as hydrogen/deuterium exchange (HDX).

Using experimental techniques sensitive to differences between hydrogen isotopes, one can monitor HDX (Englander et al., 1997; Engen et al., 2011). In the 1970s, nuclear magnetic resonance (NMR) spectroscopy was the main approach to measure HDX, leveraging the differences in magnetic properties of hydrogen and deuterium (Huang and Chen, 2014). However, HDX-NMR experiments were hindered by practical weaknesses of NMR, such as the limit on the size of proteins that could be investigated. In the 1990s, advances in mass spectrometry (MS) made this technique an interesting alternative to measure HDX. HDX-MS experiments rely on that the mass of deuterium is about twice the mass of hydrogen: deuterium uptake (i.e., the amount of deuterium incorporated in the protein) thus corresponds to an increase in mass. Some advantages of HDX-MS over HDX-NMR are that it requires only small quantities of protein sample, and that there is no strong limitation on the size of proteins that can be studied (Jaswal, 2013).

In HDX experiments, only the exchange rates of amide hydrogens (i.e., hydrogens attached to backbone nitrogens, referred to as amide nitrogens) are monitored (Engen et al., 2011); at least this represents what is most often assumed, in a slightly simplified view of the hydrogen exchange phenomenon. As a result, HDX experiments can generate at most one measurement per amino acid residue, for all amino acids of the protein, except for proline residues and for the N-terminus of the polypeptide chain (i.e., the first amino acid in the chain) because they do not possess an amide N–H group. In HDX-NMR experiments, results are acquired at the residue level (i.e., at the level of amide groups themselves), but obtaining a good coverage of the protein is very challenging. As explained in what follows, in HDX-MS experiments, results are most often acquired at the peptide level (i.e., deuterium uptake is measured for various proteolytic peptides extracted from the protein), and usually yield a good coverage of the protein. Note that, although we do not provide details on this, obtaining HDX-MS data at the residue level is feasible (Rand et al., 2009; Kan et al., 2013).

The hydrogen-exchange rate of a given amino acid can vary up to several orders of magnitude, depending on various conditions, such as solution pH and temperature (Brier and Engen, 2008). Even though this differs among amino acids, exchange rates are generally the lowest when pH is around 2.5 and temperature is around 0°C. The exchange rate of a residue in an unstructured peptide is only affected by its adjacent amino acids; this “intrinsic” exchange rate, denoted by *k*^int^, can be predicted (Bai et al., 1993; Connelly et al., 1993). On the other hand, the exchange rate of a residue in a protein is influenced by additional factors, such as its solvent accessibility and the protein's structure; therefore, this experimentally-observed exchange rate, denoted by *k*^obs^, is slower than *k*^int^ (Wei et al., 2013). To quantify the extent to which amide hydrogens are protected from being exchanged in a protein, one can define the *protection factor* of every amino acid *i* by $P_{i}=k_{i}^{int}/k_{i}^{obs}$. In HDX-NMR experiments, results are often reported as a list of (logarithms of) protection factors.

On the other hand, HDX-MS experiments produce richer information. A typical experiment starts by equilibrating a protein in H_2_O at room temperature under physiological conditions (pH 7–8). Then, the protein is diluted with excess D_2_O for the HDX to occur. At various time points, a small quantity of solution is sampled. The HDX reaction is quenched in the sample by adding acid to lower pH to 2.5, and by cooling it to 0°C. Proteins in the sample are then digested using acidic proteases (such as pepsin) that are active under quenching conditions. This proteolytic digestion generates numerous peptides, which are portions of the protein typically 6–20 amino acids in length. The sample is then introduced into a chromatography system, to separate the peptides and automatically send them for MS analysis. This analysis allows identifying the peptides generated by the proteolytic digestion and quantifying their deuterium uptake. As the digestion and MS analysis are repeated at various time points, HDX-MS experimental results are usually reported as a set of deuterium-uptake kinetic curves for various peptides (Huang and Chen, 2014).

A crucial technical aspect of HDX-MS experiments is known as *back-exchange*. This is the process by which the deuterium atoms incorporated by the peptides exchange back to hydrogens. This happens when the sample is prepared for MS analysis because all the required steps (quenching, enzymatic digestion, desalting, chromatographic separation) are performed in H_2_O solution. On the one hand, back-exchange is beneficial because it enables fast-exchanging side-chain positions to revert to hydrogens, which greatly facilitates the MS identification of peptides by limiting mass changes to amide groups (Wei et al., 2013). On the other hand, back-exchange can become detrimental if slower-exchanging amide groups start reverting to hydrogens, which means losing the information generated by the experiment (Mayne, 2016). To mitigate this problem, all experimental steps have to be performed rapidly, at low temperature.

Unfortunately, back-exchange of amide groups cannot be totally avoided, which affects several aspects of HDX-MS experiments. First, depending on the kind of performed analysis, the measurements produced by the mass spectrometer might have to be corrected for back-exchange (Engen et al., 2011). Second, because terminal positions of a polypeptide chain are more susceptible to back-exchange than other positions, the analysis of peptide-level deuterium-uptake curves has to account for it. More specifically, if the HDX experienced by a given peptide is considered as the average HDX undergone by its amino acids (as done in Section 3.1), the first two amino acids in the chain have to be ignored (Konermann et al., 2011; Huang and Chen, 2014). Indeed, after digestion, the first amino acid of the peptide becomes an amine-terminus, therefore losing its deuterium; as a result, the second amino acid usually undergoes back-exchange as well (Mayne, 2016).

### 2.2. Hydrogen exchange estimated from protein structure

Numerous theoretical models have been suggested to formalize a relationship between local and/or global structural properties of a protein and the level of hydrogen exchange it undergoes locally. However, none of these models has yet been largely accepted by the scientific community. Several of them have also shown limitations in a recent evaluation study (Skinner et al., 2012b). In this section, we mention the ideas that prevailed in the early days of the research on hydrogen exchange mechanisms, and introduce various models proposed during the past 10 years.

Early attempts to connect hydrogen-exchange mechanisms with protein structure, in the 1970s, were based on accessibility or penetration models. A common view was that solvent-accessible hydrogens located at the protein's surface would exchange rapidly, and that buried hydrogens would exchange more slowly. In other words, protection from exchange was thought to be positively correlated with atom burial or, equivalently, negatively correlated with solvent penetration in the protein matrix. However, it is now well recognized that atom burial is not the primary factor in characterizing hydrogen exchange (Konermann et al., 2011; Skinner et al., 2012b). Indeed, hydrogen-bonded amide groups at the surface can exchange as slowly as deeply-buried amide groups. A variant of this early model of hydrogen exchange based on solvent penetration became popular in the 1980s: hydrogen exchange was thought to be positively correlated with solvent accessibility surface area (SASA). Although this correlation is in general relatively weak (Skinner et al., 2012b; Radou et al., 2014), it has been observed for surface loops of non-globular proteins (Truhlar et al., 2006). This model has been used in qualitative studies of hydrogen exchange (Petruk et al., 2013), sometimes including rigidity properties for increased accuracy (Sljoka and Wilson, 2013).

To explain the fact that even solvent-exposed hydrogens can exchange very slowly, several protein properties have been investigated. For example, there have been some attempts to show that hydrogen exchange is modulated by electrostatic effects on the relative acidity of amides (Anderson et al., 2008; Avbelj and Baldwin, 2009; Hernández et al., 2009; LeMaster et al., 2009). Although this appears to be true in specific cases, in general, no correlation can be expected between protection from hydrogen exchange and changes in relative acidity of amides evaluated via electrostatic calculations (Skinner et al., 2012b). On the other hand, participation in hydrogen bonds is usually recognized as a strong determinant of protection from hydrogen exchange (Skinner et al., 2012b). However, approaches that consider only hydrogen bonding to explain protection from exchange, such as those described in Ma and Nussinov (2011) and Park et al. (2015), are not expected to generalize well. Therefore, some attempts have been made to combine several factors, such as N–H coupling constants and residue fluctuation (Brand et al., 2007).

The most successful approaches to date have been those that combine packing density with various properties related to protein dynamics. On the one hand, some approaches, such as the COREX family of tools, have attempted to link hydrogen exchange to large segmental unfolding reactions (Hilser et al., 2006; Wrabl et al., 2011; Liu et al., 2012). However, a drawback of COREX is that it heavily relies on SASA for doing so. On the other hand, other approaches have attempted to link hydrogen exchange to local interactions (Wu et al., 2009; Gogonea et al., 2010; Craig et al., 2011). Among them, the approach we have adopted in our work relies on the combined evaluation of hydrogen bonding and packing density (Vendruscolo et al., 2003; Best and Vendruscolo, 2006; Gsponer et al., 2006; Kieseritzky et al., 2006; Radou et al., 2014). It is based on a phenomenological equation approximating hydrogen-exchange protection, which is detailed in Section 3.1. Of note, there has been an attempt to predict the coefficients of this phenomenological equation from a protein's amino acid sequence (Tartaglia et al., 2007). Other methods have similarly focused on estimating structural parameters related to hydrogen exchange, directly from protein sequence (Dovidchenko et al., 2009; Lobanov et al., 2013).

## 3. Materials and methods

### 3.1. Phenomenological approximation of hydrogen exchange

As mentioned in Section 2.1, the levels of hydrogen exchange observed in different parts of a protein are known to be partly influenced by its local structure. Several theoretical models have been proposed to formalize a relationship between a protein's conformation and the corresponding hydrogen exchange (see Section 2.2). However, none of them benefits from a consensus of the scientific community, and several of them have shown limitations (Skinner et al., 2012b). Among these models, we chose the one that seemed the most promising, based on its performance in a recent comparative study (Skinner et al., 2012b) and on the number of publications in which it features (Vendruscolo et al., 2003; Best and Vendruscolo, 2006; Gsponer et al., 2006; Kieseritzky et al., 2006; Tartaglia et al., 2007; Radou et al., 2014).

The model we use to estimate hydrogen exchange from a protein's conformation relies on the definition of a phenomenological expression to approximate the protection factors (cf. Section 2.1) of the protein's residues (Vendruscolo et al., 2003). In this theoretical model, it is assumed that protection from hydrogen exchange results from the presence of hydrogen bonds involving amide groups and from the packing density of atoms around these amide groups. More precisely, the protection factor of residue *i* in conformation *C*, *P*_*i*_(*C*), is derived from the phenomenological expression

(1) $$\begin{array}{l} \ln\;P_{i}\left(C\right)=\beta^{h}N_{i}^{h}\left(C\right)+\beta^{c}N_{i}^{c}\left(C\right), \end{array}$$

where $N_{i}^{h}\left(C\right)$ is the number of hydrogen bonds formed by the amide hydrogen of residue *i*, and $N_{i}^{c}\left(C\right)$ is the number of so-called “atom contacts” (which is used to quantify packing density) involving residue *i*. Parameters β^h^ and β^c^ were estimated by fitting experimental hydrogen-exchange data from seven proteins, which lead to: β^h^ = 2 and β^c^ = 0.35 (Best and Vendruscolo, 2006).

Instead of being estimated from a single conformation, hydrogen exchange can also be estimated from a conformational ensemble. In that case, protection factors are computed as ensemble averages. Given a set of conformations, *S*, the protection factor of residue *i* with respect to *S* is derived from

(2) $$\begin{array}{l} \ln\;P_{i}\left(S\right)=\frac{1}{\left|S\right|}\underset{C\in S}{\sum }\ln\;P_{i}\left(C\right). \end{array}$$

The way hydrogen bonds and atom contacts are accounted for has changed over the years, following the evolution of the theoretical model (Vendruscolo et al., 2003; Best and Vendruscolo, 2006). Additionally, not all the details of the methodology have been published. Building on this model, we define hydrogen bonds and atom contacts in the following way:

- We only consider the hydrogen bonds maintaining secondary structure elements because they are more important than other hydrogen bonds in protecting amide groups from exchange. More specifically, only main-chain oxygens are considered as potential acceptors, when an amide nitrogen is regarded as potential donor. We count only the acceptor oxygens that are within a *cutoff* distance of 2.4 Å from the amide hydrogen. Additionally, when estimating $N_{i}^{h}\left(C\right)$, oxygens from residues *i* − 2, …, *i* + 2 are not considered as potential acceptors. This is justified by the fact that α-helices, 3_10_-helices and β-sheets are formed by N−H···O=C hydrogen bonds involving residues that are at least three positions apart in the protein's sequence.
- The number of contacts, $N_{i}^{c}\left(C\right)$, is defined as the number of heavy atoms (i.e., non-hydrogen atoms) in any residue, apart from residues *i*−2, …, *i* + 2, within a *cutoff* distance of 6.5 Å from the amide hydrogen of residue *i*. Note that these contacts are not restricted to secondary structure elements.

The residues' protection factors derived from Equation (1) can be directly compared to protection factors obtained from an HDX-NMR experiment. On the other hand, HDX-MS experiments produce deuterium-uptake curves of peptides extracted from a protein. Therefore, a similar kind of data has to be derived from the protein's structure to allow for a comparison with HDX-MS data. For that, we consider that the deuterium uptake of a residue follows pseudo-first-order kinetics (Brier and Engen, 2008; Konermann et al., 2011; Huang and Chen, 2014). Knowing that $P_{i}=k_{i}^{int}/k_{i}^{obs}$, the fraction of deuterium incorporated by residue *i* at time *t* can be expressed as

(3) $$\begin{array}{l} d_{i}\left(t\right)=1-\exp\left(-k_{i}^{obs}\;t\right)=1-\exp\left(-\left(k_{i}^{int}/P_{i}\right)\;t\right). \end{array}$$

As $k_{i}^{int}$ is known (Bai et al., 1993; Connelly et al., 1993), *d*_*i*_(*t*) can be derived from the protein's conformation by calculating *P*_*i*_. The deuterium uptake of a peptide can be considered as an average over the residues it contains. Therefore, the fraction of deuterium incorporated by peptide *j* at time *t* is

(4) $$\begin{array}{l} D_{j}\left(t\right)=\frac{1}{n_{j}}\overset{n_{j}}{\underset{i=1}{\sum }}d_{i}\left(t\right), \end{array}$$

where *n*_*j*_ is the number of residues containing an exchangeable amide hydrogen in peptide *j* (Radou et al., 2014). Note that, in addition to the N-terminal amino acid and to prolines, we systematically exclude from the average the second amino acid (even if it contains an amide group) because of back-exchange (see Section 2.1) (Konermann et al., 2011; Huang and Chen, 2014). Using Equation (4), one can obtain deuterium-uptake curves for various peptides, from any protein conformation.

### 3.2. Goodness-of-fit between structurally-derived and experimental HDX data

Using the HDX prediction model presented in Section 3.1, one can derive HDX data from a protein's conformation and compare it to the experimental HDX data. Then, assessing the goodness-of-fit between structurally-derived and experimentally-observed HDX data can be done as follows:

- When dealing with HDX-NMR data (i.e., protection factors of residues), one can obtain a histogram of differences by computing, for every residue *i*, the error $\left|\ln\;P_{i}^{der}-\ln\;P_{i}^{obs}\right|$, where $P_{i}^{der}$ is the structurally-derived protection factor and $P_{i}^{obs}$ is the experimentally-observed protection factor. This histogram can be aggregated into an average over all residues (as done in Section 4.1): $\frac{1}{n}\overset{n}{\underset{i=1}{\sum }}|\ln\;P_{i}^{der}-\ln\;P_{i}^{obs}|$, where *n* is the number of protein residues for which measurements have been obtained in the HDX-NMR experiment. Alternatively, one can compute the *R*^2^ correlation coefficient between the series ${\left\{\ln\;P_{i}^{der}\right\}}_{i=1}^{n}$ and ${\left\{\ln\;P_{i}^{obs}\right\}}_{i=1}^{n}$ (as done in Section 4.2).
- With HDX-MS data (i.e., deuterium-uptake curves of peptides), one can obtain a histogram of differences (as done in Section 4.3) by computing, for every peptide *j*, the error $\underset{t\in T}{\sum }|D_{j}^{der}\left(t\right)-D_{j}^{obs}\left(t\right)|$, where *T* is the list of experimental time points, $D_{j}^{der}\left(t\right)$ is the structurally-derived deuterium uptake at time *t*, and $D_{j}^{obs}\left(t\right)$ is the experimentally-observed deuterium uptake at time *t*. This histogram can also be aggregated into an average difference over all peptides (as done in Section 4.3).

### 3.3. Conformation providing the best fit to experimental HDX data

The question that remains is: which conformation should the HDX data be derived from to obtain a good fit to the experimentally-observed HDX data? Several studies have shown that conformations reported in the PDB (and more specifically crystal structures) do not provide good estimates for experimental HDX data (Radou et al., 2014; Devaurs et al., 2016). This can be explained by the very nature of HDX data: as it reflects the inherent flexibility of a molecule, in theory, it cannot be accurately predicted from a single conformation. Therefore, it was suggested that hydrogen exchange should be estimated from an ensemble of conformations extracted from an MD simulation, to account for the variability of a protein's structure (Best and Vendruscolo, 2006). Our previous study shows that this methodology also has limitations: better estimates of the experimental HDX data can sometimes be obtained from a single conformation extracted from a conformational ensemble produced by an MD simulation than from the whole ensemble (Devaurs et al., 2016). This shows that, in the context of the structural analysis of experimental HDX data, it is relevant to try and fit this data using a single conformation.

In this work, using computational methods that can sample protein conformations, we aim to obtain a single conformation that can help analyze the experimental HDX data collected for a protein. As PDB conformations produced by X-ray crystallography do not generally provide good estimates for experimental HDX data, they are usually not the best choice for a structural analysis of this HDX data. In spite of this, in our experiments, we systematically evaluate the goodness-of-fit achieved when comparing experimentally-observed HDX data against HDX data derived from a PDB conformation. This provides a baseline against which other methods can be compared. The two methods we evaluate in this study are MD simulations and coarse-grained conformational sampling.

#### 3.3.1. MD simulations

In this study, all MD simulations were performed with the GROMACS v4.6.5 package (Pronk et al., 2013) using the GROMOS96 (53a6) force field. A cubic box was defined with at least 9 Å of liquid layer around the protein (the exact dimensions were different for each protein), using SPC water model and periodic boundary conditions. An appropriate number of sodium (Na^+^) and chloride (Cl^−^) counter-ions were added to neutralize the system, with final concentration of 0.15 mol/L. The algorithms *v-rescale* (τ_*t*_ = 0.1 ps) and *parrinello-rhaman* (τ_*p*_ = 2 ps) were used for temperature and pressure coupling, respectively. Cutoff values of 1.2 nm were used both for van der Waals and Coulomb interactions, with Fast Particle-Mesh Ewald (PME) electrostatics. For all MD simulations, the production stage was preceded by (i) three steps of Energy Minimization (alternating steepest-descent and conjugate gradient) and (ii) eight steps of Equilibration. The Equilibration stage started with position restraints for all heavy atoms (5,000 kJ^−1^mol^−1^nm^−1^) and a temperature of 310 K, for a period of 300 ps, to allow for the formation of solvation layers. The temperature was then reduced to 280 K and the position restraints were gradually reduced. This process was followed by a gradual increase in temperature (up to 300 K). Together, these Equilibration steps represent the first 500 ps of each simulation. During the production stage, the system was held at constant temperature (300 K) without restraint. The MD simulations were run on various high-performance computers, using between 32 and 144 threads, depending on the size of the protein; the production stage lasted between 150 and 300 ns (additional protein-specific information is provided in Section 3.4). Then, we estimated HDX data as an average over the ensemble of conformations produced by a simulation. We also derived HDX data from every single conformation extracted from such a conformational ensemble.

#### 3.3.2. Structured intuitive move selector (SIMS)

In this paper, we propose a new methodology to obtain a better fit to experimental HDX data, using conformations produced by a coarse-grained conformational sampling approach. For that, we use a computational framework, called Structured Intuitive Move Selector (SIMS), that was developed to explore a protein's conformational space (Gipson et al., 2013). This framework integrates methods known as sampling-based motion-planning algorithms, initially proposed in the field of robotics to randomly explore high-dimensional spaces (Hsu et al., 1999; Şucan and Kavraki, 2010). Using these methods, exploring a protein's conformational space consists of incrementally building a graph whose nodes are conformations and whose edges represent potential transitions between them (Al-Bluwi et al., 2012; Gipson et al., 2012). SIMS follows a “coarse-grained” approach, similarly to MD-like methods using coarse-grained force fields (Davtyan et al., 2012), Monte-Carlo-based simulations (Sim et al., 2012; Boomsma et al., 2013), methods using elastic network models (López-Blanco and Chacón, 2016), or other robotics-inspired conformational sampling methods (Devaurs et al., 2013, 2015).

In SIMS, the exploration starts from a known conformation of the protein (usually, a crystal structure available in the PDB) and aims at producing new conformations by perturbing existing ones. Conformational sampling involves perturbations of the protein's structure, referred to as *protein moves*. These moves are common perturbation strategies, such as loop sampling, rigid-body motion (i.e., fix one loop's end and move the other end), random perturbation of backbone dihedral angles, and overall energy minimization. To implement these moves and calculate molecular energy, SIMS relies on the Rosetta modeling software (Das and Baker, 2008; Kaufmann et al., 2010). Additionally, SIMS involves an energy threshold to filter the conformations it generates. Note that, by varying this threshold, SIMS can be made more permissive than a typical MD simulation, with respect to the energy of the conformations it generates.

SIMS involves an internal-coordinate representation of proteins in which bond lengths and bond angles are assumed to be constant. Additionally, taking into account the planarity of peptide bonds, the associated torsion angles are restricted to their trans conformation (i.e., ω = 180°). In SIMS, a protein's conformation is represented by a vector of backbone (φ, ψ) dihedral angles. Side chains are not explicitly modeled in a conformation, but they are automatically optimized by Rosetta when a move is performed. As a result, a protein composed of *N* + 1 residues is modeled with 2*N* degrees of freedom. Such a coarse-grained model has long been shown to provide a good approximation of a protein's behavior (Levitt, 1976).

In SIMS, proteins are decomposed into *fragments* on which moves are applied. Fragments are specific sets of residues that can be defined automatically, based on secondary structure, or that can be chosen by a domain expert. A fragment can be a protein domain, a single secondary structure element (or several of them), a single residue, or several (non-necessarily contiguous) residues. Using these fragments, one can favor the sampling of specific regions of the protein during conformational exploration. Indeed, based on how flexible some regions are expected to be, fragments are assigned probabilities to be perturbed during conformational sampling (Gipson et al., 2013). These probabilities can reflect available expert knowledge of the protein, reported experimental data (such as B factors) or predicted data resulting from a computational analysis (Fox et al., 2011). In some of the experiments presented here, we use discrepancies between experimentally-observed and structurally-derived HDX data to define these probabilities and therefore guide conformational exploration.

Our experimental methodology can be summarized as follows: First, we use SIMS to perform a conformational exploration starting from the crystal structure of a protein, without using any sampling bias. From the ensemble of conformations generated by SIMS, we determine which conformation provides the best estimates of the HDX data, using Equations (1)–(4). If a good fit is obtained, no additional run of SIMS is performed. If the goodness-of-fit is too low, we run SIMS again, using the largest discrepancies between experimentally-observed and structurally-derived HDX data as a sampling bias: the protein regions where these discrepancies are the largest are assigned higher probabilities to be sampled. We repeat this process a given number of times, or stop when a conformation providing a good fit to the HDX data is obtained.

A single run of SIMS lasted 24 h and was performed on four threads of a 3.6 GHz Intel i7-4790 quad-core CPU. For small proteins, we ran SIMS only once, but for the largest one, we ran SIMS five consecutive times (see Section 4 for more details). For comparison, if the aforementioned MD simulations were run on the same computer, 24 h of computation would yield only 5–15 ns of simulation, therefore requiring days to weeks for a whole simulation, depending on protein size.

### 3.4. Studied proteins and experimental HDX data

First, we use two small proteins (CI2 and Im7) to illustrate the concepts involved in our methodology. As they have been extensively studied, they represent useful benchmarks. Then, we analyze two medium-size proteins (SN and C3d) that represent more challenging targets for our methodology. Note that we consider two kinds of HDX data: HDX-NMR for CI2, Im7 and SN; and HDX-MS for C3d.

#### 3.4.1. Chymotrypsin inhibitor 2 (CI2)

We consider a truncated form of chymotrypsin inhibitor 2 (CI2) composed of 64 amino acids (PDB 1TM1), where residue 1 corresponds to residue 20 of the full protein. The main secondary structure elements of CI2 are the following: residues Val13 to Asp23 form an α-helix; residues Gln28 to Pro33 and residues Arg46 to Val51 form two β-strands. As a simple system for folding studies, CI2 has been the subject of several HDX-NMR experiments (Itzhaki et al., 1997; Neira et al., 1997). Protection factors for more than half of CI2's residues have been reported. However, as done in other studies (Best and Vendruscolo, 2006), we only use the protection factors associated with local hydrogen-exchange mechanisms characteristic of CI2's native state. Therefore, we only consider the following 14 residues (whose protection factors are given in parentheses): Leu8 (8.1), Val9 (9.9), Val13 (7.2), Ala16 (7.1), Lys17 (6.6), Lys18 (8.2), Gln22 (9.5), Ala27 (6.7), Gln28 (8.2), Asp52 (8.5), Asn56 (8.4), Ala58 (9), Gln59 (10.5), Val63 (7.4). Note that these protection factors are given as ln *P*, based on published exchange rates (Itzhaki et al., 1997). Three trajectories of CI2 were obtained by running MD simulations with a 150 ns production stage.

#### 3.4.2. Bacterial immunity protein Im7

The bacterial immunity protein Im7 is a single-domain α protein composed of 86 residues (PDB 1AYI). Im7's native state comprises four α-helices: residues Glu12 to Lys24 (I), residues Asp32 to Thr45 (II), residues Thr51 to Tyr56 (III), and residues Glu66 to Asn79 (IV). Helices I and II form an N-terminal helical hairpin, and helix IV is located along the open end of this hairpin. Im7 has been shown to fold through an on-pathway intermediate whose structure is significantly different from that of its native state (Gorski et al., 2004). In this non-native state, helices I, II and IV are conserved, but helix III is not formed. A computational analysis of this intermediate state has shown that helices I, II, and IV are not organized as they are in the native state (Gsponer et al., 2006). This analysis was based on protection factors of 26 residues, obtained via an HDX-NMR experiment aimed at characterizing Im7's folding intermediate (Gorski et al., 2004). Here, we consider the same residues (whose protection factors are given in parentheses): Asp9 (8), Tyr10 (9.2), Thr11 (10.2), Val16 (11.5), Gln17 (11.6), Leu18 (11.2), Glu21 (8.5), Glu23 (6), Leu37 (6.1), Leu38 (7.5), Phe41 (6), Val42 (10.3), Leu53 (3.5), Ile54 (3.6), Tyr55 (4.4), Tyr56 (5.6), Gly67 (8.1), Val69 (8.8), Ile72 (9), Lys73 (9.5), Glu74 (9), Trp75 (8.8), Arg76 (9.9), Ala77 (9.8), Ala78 (8), Lys85 (6.8). These protection factors are given as ln *P*, based on published exchange rates (Gorski et al., 2004). Three trajectories were obtained by running MD simulations with a 200 ns production stage.

#### 3.4.3. Staphylococcal nuclease (SN)

Micrococcal nuclease, or Staphylococcal nuclease (SN), is a mixed α/β protein composed of 149 amino acids organized in two domains (PDB 1SNO). The first domain (residues 1–98) belongs to the oligonucleotide/oligosaccharide-binding-fold (or OB-fold) superfamily. It consists of a five-stranded β-barrel with Greek key topology, capped by an α-helix (residues Gly55 to Glu67) located between the third and fourth strands. The five β-strands are: residues Lys9 to Ala17, residues Thr22 to Tyr27, residues Gln30 to Leu36, residues Ile72 to Phe76, residues Gly88 to Ala94. The second domain (residues 99–149) contains two α-helices: residues Val99 to Arg105, and residues Glu122 to Lys134. SN also contains two minor β-strands. HDX-NMR experiments have been performed on a double mutant of SN with similar structure but increased stability, to characterize its native state (Skinner et al., 2012b). This allowed measuring hydrogen-exchange rates for most residues and deriving corresponding protection factors. Here, we use 100 of these protection factors: residues of the N and C terminals that are missing from the crystal structure (PDB 1SNO) are not considered. Note that protection factors were reported as log_10_*P*, instead of ln *P* (Skinner et al., 2012b).

#### 3.4.4. Complement protein C3d

C3d is a fragment of the complement component C3 (Nagar et al., 1998; Hammel et al., 2007). It is a single-domain α protein composed of 297 residues (PDB 2GOX), where residue 1 corresponds to residue 991 of the full C3 molecule. C3d contains twelve α-helices and five 3_10_-helices that are organized into an α-α barrel where most consecutive helices alternate between the inside and the outside. Based on previous notations (Nagar et al., 1998), the core of the barrel consists of the following six parallel α-helices: α_1_ (residues Glu22 to Thr41), α_3_ (residues Thr86 to Leu102), α_5_ (residues Lys149 to Ala164), α_8_ (residues Ser196 to Met209), α_10_ (residues Gln236 to Leu253), and α_12_ (residues Ser278 to Asp295). It is surrounded by another set of six parallel helices (running anti-parallel to those of the core) comprising one 3_10_-helix, T_1_ (residues Ala7 to Leu13), and five α-helices: α_2_ (residues Leu49 to Arg70), α_4_ (residues Ser107 to Lys121), α_7_ (residues Ser174 to Asn189), α_9_ (residues Pro215 to Thr223), and α_11_ (residues Phe256 to Gln269). In previous work, we performed an HDX-MS experiment and several MD simulations on C3d, to characterize its native state (Devaurs et al., 2016). In this paper, as in our previous study, we use the deuterium-uptake data obtained for 81 peptides extracted from C3d.

## 4. Results

We now report the results we have obtained for the four proteins introduced in Section 3.4. First, a comparative analysis of CI2 and Im7 sheds light on the issues encountered when trying to fit experimental HDX data with the different methods presented in Section 3.3. Then, we examine two medium-size proteins: SN and C3d.

### 4.1. Chymotrypsin inhibitor 2 vs. bacterial immunity protein Im7

Our first set of results aims at highlighting differences and similarities that exist between two possible scenarios: (i) the case where the HDX data and the crystal structure describe the same state of the protein, and (ii) the case where they describe two different states. As mentioned in Section 3.4.1, the HDX-NMR data (i.e., protection factors of residues) obtained for CI2 is characteristic of its native state, whose structure has been described (PDB 1TM1). On the contrary, the HDX-NMR data gathered for Im7 is known to characterize a non-native folding intermediate (see Section 3.4.2) that is structurally different from Im7's native state (PDB 1AYI).

**Figure 1:**
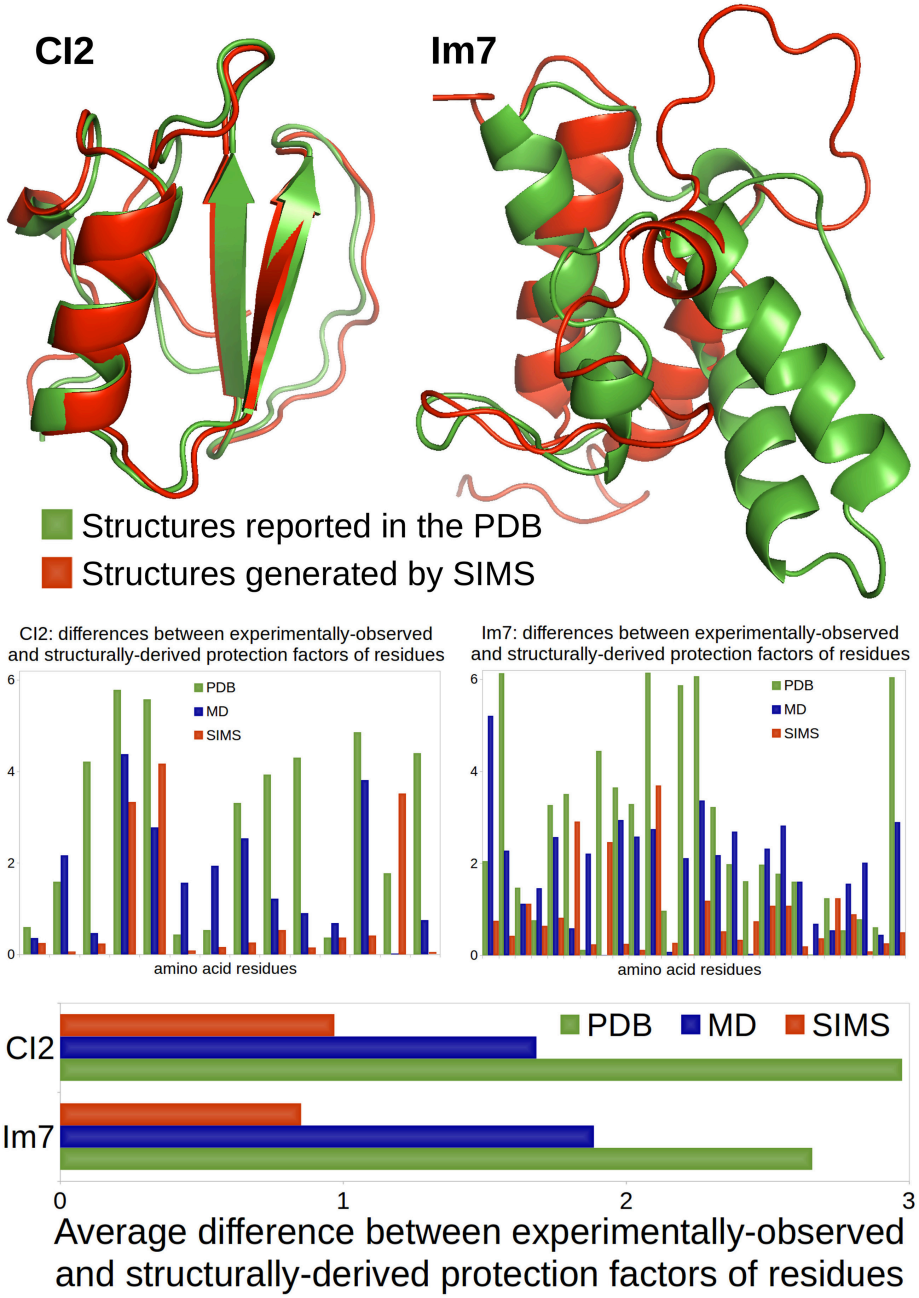
**Comparison of a case where HDX data and crystal structure describe the same state (CI2) and a case where they describe different states (Im7)**. CI2's HDX data is characteristic of its native state, described in the PDB. Im7's HDX data characterizes a non-native folding intermediate that structurally differs from the native state reported in the PDB. All conformations are represented using the ribbon model: conformations reported in the PDB are colored in green; conformations produced by SIMS that provide the best estimates of the experimental HDX data are colored in red. The bar chart at the bottom shows the average difference (across residues) between experimentally-observed and structurally-derived HDX data (i.e., protection factors), when deriving this data using conformations in the PDB (green), conformations produced by MD which best fit the HDX data (blue), or conformations generated by SIMS which best fit the HDX data (red). The bar charts in the middle show these differences for all residues separately.

The comparison between these two scenarios is illustrated in Figure 1. The native conformations of CI2 and Im7, as reported in the PDB, are depicted in green using the ribbon model. The bar charts show that the HDX data derived from the PDB conformations (i.e., the crystal structures reported in the PDB) using Equation (1) does not match well the experimentally-observed HDX data: the average difference between structurally-derived and experimentally-observed protection factors (see Section 3.2) is close to 3. Although this is not surprising in the case of Im7 (because the HDX data and the crystal structure describe different states), it is important to note that the HDX estimates are equally bad in the case of CI2 (even though the HDX data and the crystal structure describe the same state).

For both CI2 and Im7, we performed three MD simulations. We observe that, as suggested in Radou et al. (2014), deriving HDX data from the ensemble of conformations extracted from each MD simulation leads to a better fit to experimentally-observed HDX data than if the PDB conformation is used. However, it also appears that the best fit is usually obtained with a single conformation selected within these MD ensembles, which confirms our previous results on C3d (Devaurs et al., 2016). The bar charts in Figure 1 show the differences between the experimentally-observed and structurally-derived protection factors, when deriving these protection factors from the MD conformation providing the best fit. It is clear that using this MD conformation yields a better fit to the experimental data than using the PDB conformation, but not drastically. In the case of Im7, the limited improvement was expected: our MD simulations were meant to sample the native state; they were not long enough to observe a transition to the folding intermediate. Even in the case of CI2, we will show that better results can be obtained.

We used SIMS to sample the conformational space of CI2 and Im7, starting the exploration from their PDB conformations, without any bias. From the sets of conformations generated by SIMS, we extracted the conformation yielding the best fit between structurally-derived and experimentally-observed HDX data. The bar charts in Figure 1 show differences between experimentally-observed and structurally-derived protection factors, when deriving them from the SIMS conformation with the best fit. This conformation yields a significantly better fit to experimental HDX data than the PDB and MD conformations. The SIMS conformations for CI2 and Im7 are depicted in red using the ribbon model in Figure 1. In the case of CI2, the SIMS conformation is very similar to the PDB conformation: differences occur mostly in side-chain positions and not in backbone structure. This was expected because the HDX data and the crystal structure describe the same state. This also highlights the strong impact that even small structural variations can have when estimating protection factors with Equation (1). In the case of Im7, the SIMS conformation providing the best fit to the experimental HDX data is significantly different from the PDB conformation, which confirms that the HDX data and the crystal structure describe different states.

### 4.2. Staphylococcal nuclease

A recent evaluation study of various models for deriving hydrogen exchange from a protein's structure involved HDX-NMR data gathered for SN (see Section 3.4.3) (Skinner et al., 2012b). The study concludes that, at least for SN, none of the evaluated models can produce HDX data that fits well the experimentally-observed HDX data. The best results are achieved by the model based on Equation (1), with a correlation coefficient *R*^2^ = 0.51 between the structurally-derived and experimentally-observed protection factors. That study follows the methodology in Best and Vendruscolo (2006), estimating the protection factors of SN's residues using an ensemble of conformations extracted from an MD simulation. However, that study does not consider estimating HDX data from the PDB conformation alone, using Equation (1). Interestingly, using this PDB conformation, we obtained a correlation coefficient *R*^2^ = 0.69 between the structurally-derived and experimentally-observed protection factors (see Section 3.2). Note that better results can be achieved with our novel methodology.

**Figure 2:**
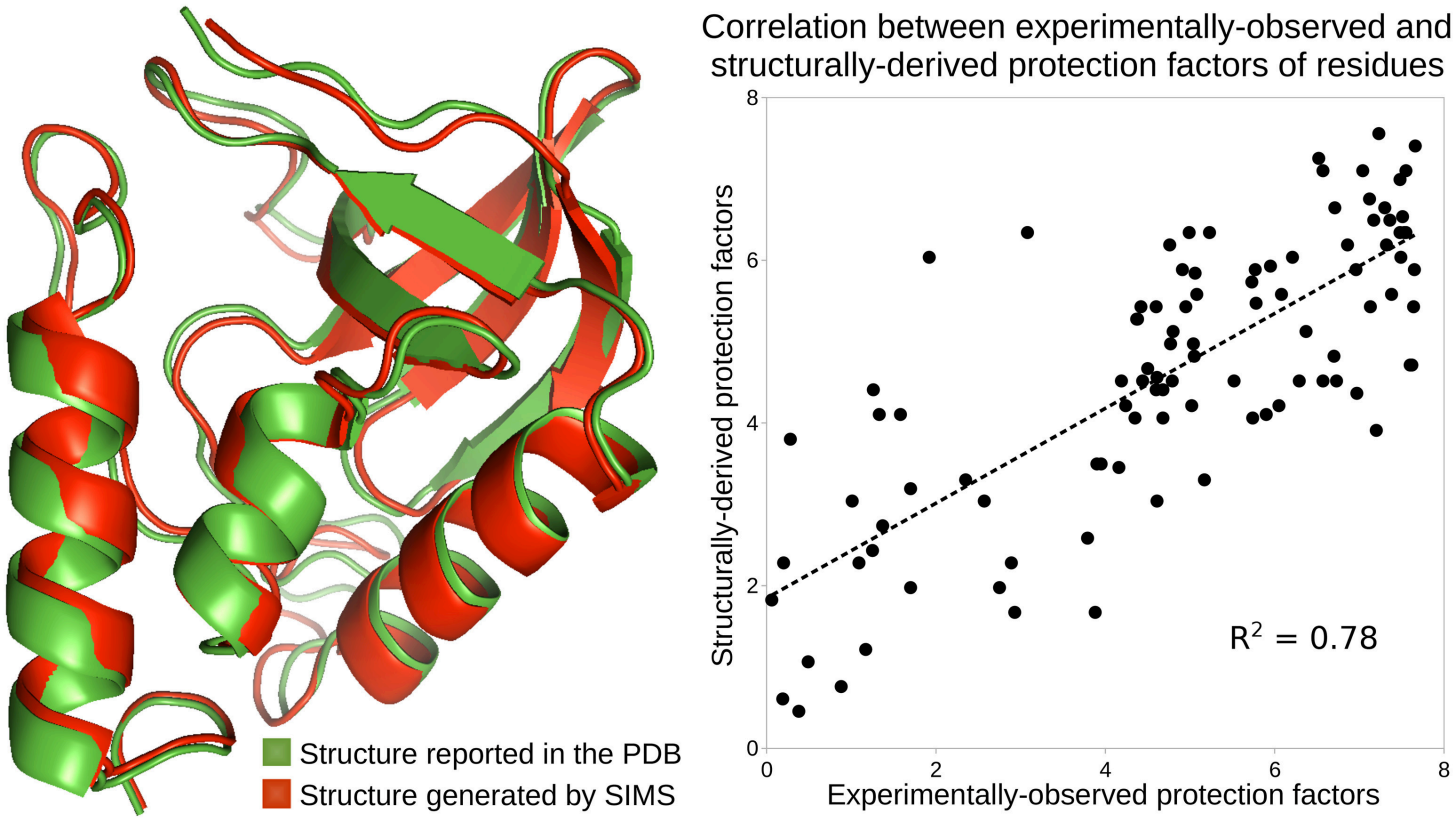
**Analysis of the native state of SN**. Conformations of SN are depicted using the ribbon model: the conformation reported in the PDB, in green, and the conformation generated by SIMS which provides estimates of protection factors that best fit the experimental HDX data, in red. The plot shows the correlation between the experimentally-observed HDX data and the HDX data derived from the SIMS conformation. The correlation coefficient is 0.78.

We used SIMS to explore the conformational space of SN, starting from its PDB conformation, without introducing any bias. From the ensemble of conformations generated by SIMS, we extracted the conformation providing estimates of protection factors that best fit the experimental HDX data. This yields a correlation coefficient *R*^2^ = 0.78 between the structurally-derived and experimentally-observed protection factors, as shown in Figure 2. Importantly, the SIMS conformation is very similar to the PDB conformation: only small structural differences are observed at the backbone level (see Figure 2). This confirms that the HDX data and the crystal structure both describe SN's native state.

### 4.3. Complement protein C3d

**Figure 3:**
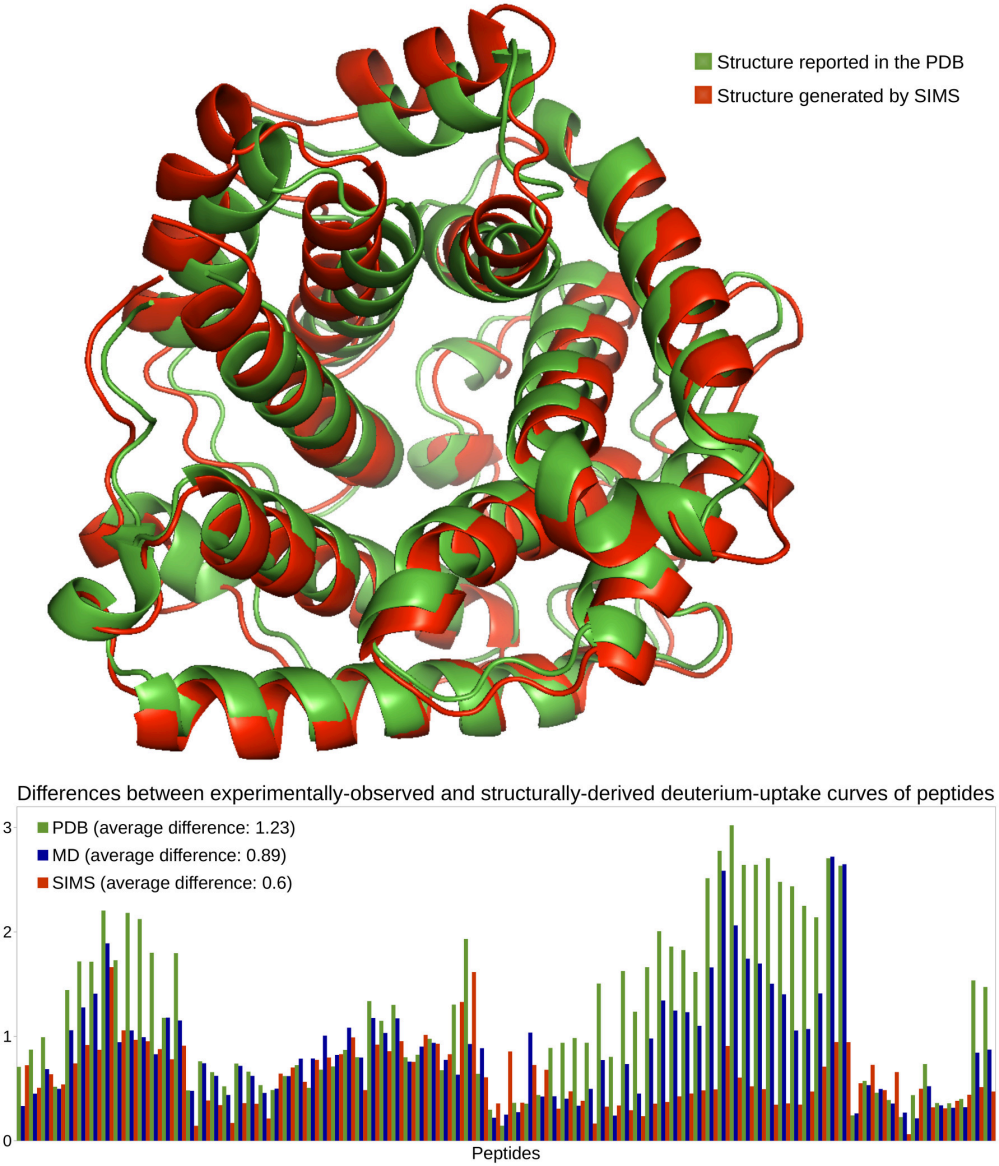
**Analysis of the native state of C3d**. Conformations of C3d are depicted using the ribbon model: the conformation reported in the PDB, in green, and the conformation generated by SIMS which provides estimates of deuterium-uptake curves that best fit the experimental HDX data, in red. The plot shows differences between the experimentally-observed and structurally-derived deuterium-uptake curves, for all peptides, when deriving this data from the PDB conformation (green), the MD conformation (blue) or the SIMS conformation (red). The legend also includes the average differences across all peptides.

In previous work (Devaurs et al., 2016), we performed an HDX-MS experiment on C3d and obtained deuterium-uptake curves for 86 peptides. As in that previous study, we restrict the current analysis to the 81 peptides whose data is the most reliable. This HDX data is expected to describe the native state of C3d when present alone in solution. However, once again, deuterium-uptake curves derived from the PDB conformation of C3d (PDB 2GOX) using Equations (1)–(4) do not fit well the experimental data (see Figure 3). The average difference between the experimentally-observed and structurally-derived deuterium-uptake curves across all peptides is 1.23 (see Section 3.2). Discrepancies are especially significant in the region of C3d comprising residues Met191 to Ala242. As shown in Devaurs et al. (2016), this is not due to structural differences between the native state of C3d and the conformation observed during the HDX-MS experiment, but rather to the limitations of predicting HDX data using crystal structures.

We carried out three MD simulations to sample the variability of C3d's native state (Devaurs et al., 2016). Using the ensemble of conformations extracted from each simulation allows deriving deuterium-uptake curves of peptides that fit the experimental data better than when using the PDB conformation. However, an important conclusion of our previous study is that: using a single conformation extracted from these MD ensembles produces even better results (Devaurs et al., 2016). The conformation providing the estimates of deuterium-uptake curves that best fit the experimental HDX data is referred to as the MD conformation. It yields a decrease in the average difference (0.89) between structurally-derived and experimentally-observed HDX data. Despite the improvement in goodness-of-fit, large discrepancies remain (see Figure 3), especially in the region [Met191-Ala242] of C3d (Devaurs et al., 2016).

To sample C3d's conformational space more extensively, we carried out the following iterative process with SIMS: using the PDB conformation as input, we ran SIMS once without introducing any bias; then, we ran SIMS four times, using the discrepancies between structurally-derived and experimentally-observed HDX data as a sampling bias. This bias is introduced in the following way: at the end of each run, we select the conformation generated by SIMS providing estimates of deuterium-uptake curves that best fit the experimental HDX data, and we determine the regions of C3d where discrepancies are the largest; then, in the following run, these regions are assigned higher probabilities to be sampled (cf. Section 3.3.2). This SIMS-based iterative process generated a conformation providing estimates of deuterium-uptake curves that fit well the experimental HDX data (see Figure 3). Using this SIMS conformation, the average difference between the experimentally-observed and structurally-derived deuterium-uptake curves across all peptides decreases to 0.6. Importantly, the SIMS conformation is very similar to the PDB conformation: all the helices forming the α-α barrel are conserved; only two short helices have unfolded. The α-α barrel of the SIMS conformation (radius of gyration: 19 Å) is only slightly wider than the α-α barrel of the PDB conformation (radius of gyration: 18 Å). This confirms that the HDX data and the crystal structure both describe C3d's native state. It also confirms that the native state of C3d is relatively stable, with little flexibility recorded by the HDX-MS experiment. Finally, note that using the SIMS conformation can provide an improved structural analysis of C3d, by refining the HDX data from the peptide level to the residue level (Devaurs et al., 2016).

## 5. Discussion

We chose to include in our methodology the phenomenological approximation of hydrogen-exchange protection, as expressed by Equation (1), because it seemed to be the most promising HDX prediction model. Indeed, it performed best at predicting experimental HDX data, when compared to several other models (Skinner et al., 2012b). Even though the goodness-of-fit achieved with this model was not impressive (Skinner et al., 2012b), our work demonstrates how it can be used successfully.

Our results clearly show that using the conformation of a protein as reported in the PDB does not provide good estimates of experimental HDX data. This confirms what was observed in previous similar studies (Radou et al., 2014; Devaurs et al., 2016). This was also indirectly acknowledged when this HDX prediction model was first proposed (Vendruscolo et al., 2003). In an attempt to consider structural dynamics, it was suggested that HDX data should be derived as an average over an ensemble of conformations produced by an MD simulation (Best and Vendruscolo, 2006).

We have indeed observed that computing an average over an MD ensemble provides better estimates of experimental HDX data than using a single PDB conformation. However, as shown in our previous work (Devaurs et al., 2016), this study confirms that using a single conformation carefully extracted from the MD ensemble usually provides even better estimates. In other words, the MD conformation that provides the best estimates within the MD ensemble performs generally better than the whole ensemble. Note that we do not claim that this MD conformation constitutes a better representation of a protein's state than a PDB conformation or an MD ensemble. In theory, the best estimates for experimental HDX data would be obtained by computing an average over an ensemble of conformations best representing a protein's state and its inherent flexibility. However, in the same way as estimates derived from two similar conformations can significantly differ, estimates derived from two similar ensembles can be very different. In practice, it is thus more convenient to generate many conformations and select the one providing the best estimates than to find the best conformational ensemble.

The fact that numerous conformations have to be generated in order to obtain good estimates of experimental HDX data, and that a PDB conformation is not enough, is also linked to weaknesses of the HDX prediction model based on Equation (1). The first limitation of this model is its lack of robustness: it is very sensitive to small variations in the protein structure. As well illustrated by the case of CI2, two conformations that are very similar at the backbone level and present differences only in their side-chain conformations can produce very different HDX estimates. The second limitation of this model is that it only partially reflects the mechanisms underlying hydrogen exchange. For example, it does not consider any dynamic aspect of proteins. Therefore, it could be interesting to develop a more accurate model by accounting for additional structural and dynamic properties of proteins (Skinner et al., 2012a). Since such a model has not been proposed yet, we believe it is best to compensate for the weaknesses of the current model by performing conformational sampling.

## 6. Conclusion

When performing a structural analysis of HDX data collected for a protein, a premise to an accurate analysis is to use a conformation that matches this data. Several studies, including ours, show that crystal structures reported in the PDB are not a good choice because they often provide bad estimates of experimental HDX data. Because HDX data reflects the inherent flexibility of a protein, a conformational ensemble should ideally provide better estimates than a single conformation. However, our work has shown that this is not always the case with a conformational ensemble produced by an MD simulation. Therefore, it is perfectly justified to try and fit experimental HDX data using a single conformation. In this paper, we have shown that this can be done using a coarse-grained conformational sampling tool to explore a protein's conformational space. The specific tool we used, called SIMS, yields a conformational ensemble from which one can extract a conformation providing a good fit to the experimental HDX data. Note that we do not claim that a conformation produced by SIMS is a better representation of a protein's state than its crystal structure. Besides the improved accuracy, another advantage of using SIMS is its efficiency: a conformation providing a good fit to experimental HDX data can be obtained at a fraction of the computational cost of running a traditional MD simulation. Finally, we believe that other conformational sampling methods could produce similar results, in terms of accuracy and efficiency. The achievement of our study mostly consists of revealing the technicalities that must be addressed for such methods to be successful.

Our methodology relies on the use of an HDX prediction model defining how to derive HDX data from a protein's structure. This model is based on a phenomenological approximation of the protection factors of a protein's residues. Despite its limitations, this model enables our methodology to successfully produce a conformation fitting the experimental HDX data. Another interesting benefit of this model is that, besides the validation of experimental HDX data, in the case of HDX-MS experiments, it offers the possibility to refine the HDX data from the peptide level to the residue level (Radou et al., 2014; Devaurs et al., 2016). This has the potential to enhance applications of the HDX-MS technique (Pirrone et al., 2015).

As part of our future work, we intend to apply our methodology to larger proteins, to evaluate its scalability. Since coarse-grained conformational sampling scales better than MD, we expect our methodology to be even more beneficial with large proteins. Additionally, we plan to investigate several useful applications of this work. First, as demonstrated with Im7, our method can be used to obtain a structural model of a non-native state of a protein when only its native state is described in the PDB and only HDX data is available for this non-native state. Second, although we applied our method only to cases where the experimental HDX data was expected to characterize a single protein conformation because a single conformer was assumed to be present in solution, it could be applied to more complex cases, where several conformers are involved. Indeed, if structurally-derived HDX data better fits experimentally-observed HDX data when deriving it from a small set of structurally-different conformations (i.e., two or three, or a handful of conformations) than when deriving it from a single conformation, we can suspect that several protein conformers are present together in solution.

## Author contributions

DR, JL, LK, MM suggested the initial idea behind this work. DD conceived the study, including the methodological choices and the selection of proteins, and implemented the scripts required for the analysis. MP performed the HDX-MS experiment. DA performed the MD simulations. DD carried out the analysis of the results and wrote the paper. DA, DR, JL, LK, MM, MP reviewed and commented on the paper.

## Funding

This work was supported in part by the National Science Foundation under Grant CCF 1423304, as well as the National Institutes of Health under Grants R21CA209941, AI068730, and AI030040. Computational simulations were run on equipment supported in part by the Data Analysis and Visualization Cyberinfrastructure funded by NSF under Grant OCI 0959097, on equipment supported by the Cyberinfrastructure for Computational Research funded by NSF under Grant CNS 0821727, as well as on equipment supported in part by the Big-Data Private-Cloud Research Cyberinfrastructure MRI-award funded by NSF under grant CNS 1338099 and by Rice University.

### Conflict of interest statement

The authors declare that the research was conducted in the absence of any commercial or financial relationships that could be construed as a potential conflict of interest.
